# Comparative Transcriptome Analysis of Walnuts (*Juglans regia* L.) in Response to Freezing Stress

**DOI:** 10.3390/plants14193089

**Published:** 2025-10-07

**Authors:** Lin Chen, Juntao Wang, Qi Zhang, Taoyu Xu, Zhongrui Ji, Huazheng Hao, Jing Wang, Gensheng Shi, Jian Li

**Affiliations:** 1 The Industrial Crop Institute, Shanxi Agricultural University, Taiyuan 030031, China; linchen@sxau.edu.cn (L.C.); xixi4426918@126.com (Q.Z.); 15513697286@163.com (T.X.); jzsjzr@163.com (Z.J.); nkshhz@163.com (H.H.); m13860442364@163.com (J.W.); ll7390903@126.com (G.S.); 2 College of Horticulture, Shanxi Agricultural University, Jinzhong 030801, China; 15038579994@163.com

**Keywords:** walnuts, freezing stress, transcriptome, flavonoids, CBF

## Abstract

Walnuts (*Juglans regia* L.) are an economically important woody crop, but spring frost poses a serious threat to their growth and productivity. However, the molecular mechanisms underlying walnut responses to freezing stress remain largely unknown. In this study, transcriptome analyses were performed on cold-tolerant and cold-sensitive walnut varieties subjected to freezing stress. A total of 9611 differentially expressed genes (DEGs) responsive to freezing stress were obtained, of which 2853 were common up-regulated and 2880 were common down-regulated in both varieties. Kyoto Encyclopedia of Genes and Genomes (KEGG) enrichment analysis revealed 15 significantly enriched pathways in both varieties, including flavonoid biosynthesis. A simplified walnut flavonoid biosynthesis pathway was constructed, encompassing 36 DEGs encoding 13 key enzymes, demonstrating that flavonoid biosynthesis in walnut is significantly activated under freezing stress. Furthermore, weighted gene co-expression network analysis (WGCNA) identified a regulatory network centered on the *JrCBF* genes and uncovered 34 potential interacting genes. Collectively, these findings provide novel insights into the molecular responses of walnut to freezing stress and establish a foundation for elucidating the mechanisms underlying walnut cold tolerance.

## 1. Introduction

Walnut (*Juglans regia* L.) is an economically important woody plant, renowned for its nutritious edible nut rich in unsaturated fatty acids, vitamins and minerals [1]. They are also a high-quality source of timber and are widely cultivated worldwide [2]. China has the largest walnut planting area in the world and is a major contributor to global walnut production. However, walnut cultivation and distribution are increasingly threatened by abiotic stresses, particularly cold stress [3].

Cold stress is one of the most important limiting factors that restricts plant productivity and geographical distribution around the world [4]. Cold stress can be classified as chilling (0–15 °C) and freezing (<0 °C) stresses [5]. The freezing stress severely threatens the growth and productivity of walnuts. For instance, prolonged low temperatures at the overwintering stage can lead to branch dieback, while the occurrence of spring frost, especially in the northern growing regions of China, damages the new shoots, buds, and flower organs of walnuts, causing a significant decrease in productivity [6,7]. Given the increasing frequency of extreme cold events, improving the freezing tolerance of walnuts is crucial for stable nut production.

Cold stress imposes a series of physiological and biochemical challenges on plants, including disruption of cell membrane fluidity and stability, excessive accumulation of reactive oxygen species (ROS), photosynthetic inhibition, and disruption of metabolic homeostasis [8]. To cope with cold stress, plant species have evolved various physiological and molecular mechanisms to increase cold tolerance, such as cellular membrane lipid remodeling, accumulation of protective substances, and activation of ROS scavenger systems [9,10]. These physiological responses are tightly regulated by a complex network of molecular mechanisms, among which the CBF (C-repeat binding factors)-COR (cold-regulated) pathway is the most well-characterized and conserved in plants [11,12,13]. In *Arabidopsis thaliana*, CBF transcription factors include *CBF1*, *CBF2*, and *CBF3*, which activate the expression of *COR* genes by binding to the CRT/DRE (C-repeat/dehydration-responsive element) cis-elements, thereby initiating the cold response of plants, including enhanced membrane stability, osmotic adjustment, and ROS scavenging [14,15,16,17]. In *J. regia*, eight *JrCBF* genes have been identified in the genome of the accession ‘Zhongmucha-1’, among which *JrCBF1* and *JrCBF2* quickly respond to low-temperature stress [18]. Despite this progress, their regulatory networks and downstream target genes have not been systematically elucidated.

RNA sequencing (RNA-seq) provides comprehensive and detailed information for mining functional genes, quantifying gene expression levels, and revealing stress responses, and is widely used in the study of walnut biotic and abiotic stress responses [19,20,21,22,23]. Recent investigations in walnuts show that RNA-seq has been used to reveal the walnut’s response to low-temperature stress of 4 °C [24,25]. However, there were few reports on the freezing response of walnuts.

To understand the complex regulation mechanisms of freezing tolerance in walnuts, a comprehensive transcriptomic analysis of two walnut varieties with contrasting cold tolerance under freezing stress was conducted. Furthermore, weighted gene correlation network analysis (WGCNA) was employed to construct the co-expression network of *JrCBFs*. The findings of this study will not only advance our understanding of the molecular mechanisms underlying the walnut’s freezing response but also provide valuable information for breeding cold-tolerant cultivars.

## 2. Results

### 2.1. Overview of Transcriptome Data

**Figure 1:**
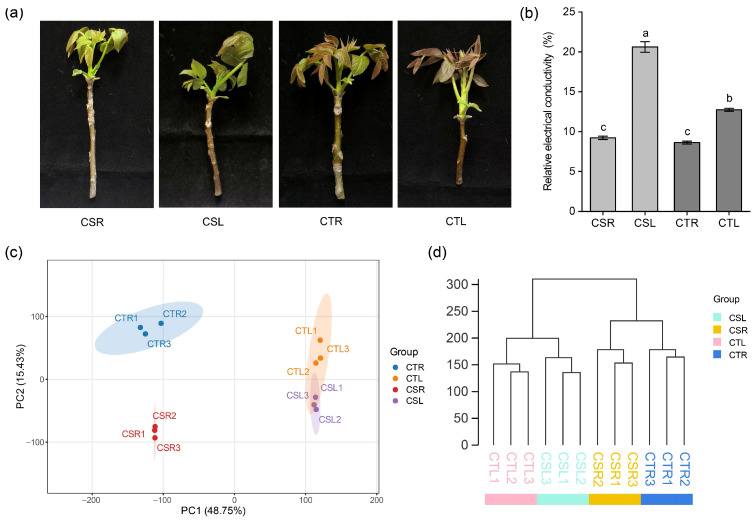
Effects of freezing treatment and transcriptome analysis in two contrasting walnut varieties. (**a**) Phenotypic responses of the cold-sensitive variety ‘Qingxiang’ (CS) and the cold-tolerant variety ‘Chandler’ (CT) after room temperature and low temperature (−2 °C) treatment; (**b**) Relative electrical conductivity (REC) of leaves from new shoots after freezing or room temperature treatments. Different letters represent a statistically significant difference between treatments determined by Duncan’s multiple range test (*p* < 0.05); (**c**) Principal component analysis (PCA) showing transcriptional variation among different treatment groups; (**d**) Hierarchical cluster dendrogram based on FPKM values of all expressed genes. CSR, the CS room temperature treatment group; CSL, the CS low-temperature treatment group; CTR, the CT room temperature treatment group; CTL, the CT low-temperature treatment group.

New shoots from the cold-sensitive variety ‘Qingxiang’ (CS) and the cold-tolerant variety ‘Chandler’ (CT) were collected and subjected to either low-temperature treatment at −2 °C or control treatment at room temperature (20 ± 1 °C) for 12 h (Figure 1a) [26]. Relative electrical conductivity (REC) was measured to assess electrolyte leakage as an indicator of cell membrane damage. As shown in Figure 1b, no significant difference in REC values was observed between CS and CT under room temperature conditions. However, the REC value of CS was significantly higher than that of CT after freezing treatment, indicating greater membrane damage in CS and confirming that CT exhibits stronger cold tolerance.

To further investigate the molecular basis of this difference, a total of 12 cDNA libraries were constructed for RNA-seq, with three biological replicates per treatment group. These libraries were divided into four groups: the CS room temperature treatment group (CSR), the CS low-temperature treatment group (CSL), the CT room temperature treatment group (CTR), and the CT low-temperature treatment group (CTL). RNA-seq generated a total of 86.5 Gb of clean data. The GC content ranged from 44.79% to 45.95%, with Q20 values exceeding 96.5% and Q30 values above 89.2%, demonstrating high sequencing quality. Alignment to the reference genome revealed that 94.92–97.47% of reads were successfully mapped, of which 87.08–92.91% were uniquely aligned. Furthermore, 93.48–97.42% of mapped reads were distributed within exon regions (Table S1).

Principal component analysis (PCA) revealed a clear separation among the different treatment groups, with biological replicates from each group clustering tightly together (Figure 1c). The first two principal components accounted for 64.18% of the total variance, highlighting the major sources of transcriptional divergence. Notably, the distinct separation between the low-temperature treatment groups (CSL, CTL) and the room temperature control groups (CSR, CTR) demonstrated that low-temperature stress exerted a strong influence on global gene expression profiles in both varieties. Similarly, hierarchical clustering analysis showed that biological replicates within the same treatment group were highly consistent (Figure 1d). This tight grouping indicates high similarity in gene expression patterns among replicates and underscores the reliability and reproducibility of the transcriptome data. Taken together, these results confirm that the RNA-seq datasets are of high quality and suitable for subsequent analysis.

### 2.2. Analysis of Differentially Expressed Genes

**Figure 2:**
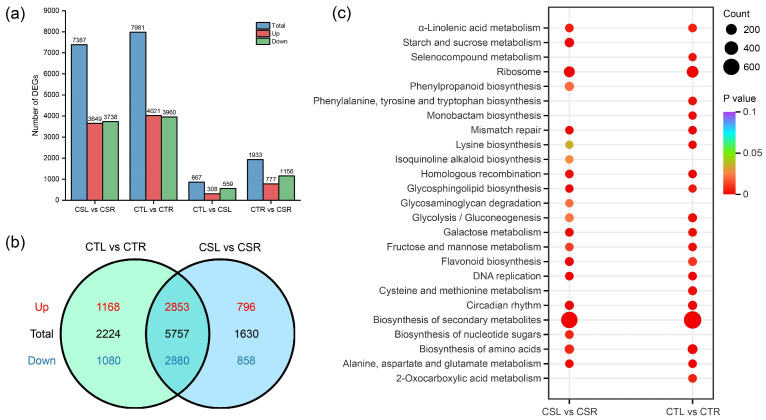
Differentially expressed gene (DEG) analysis under freezing stress in walnut. (**a**) Number of DEGs in each comparison group; (**b**) Venn diagram showing the overlap of DEGs between CSL vs. CSR and CTL vs. CTR; (**c**) KEGG enrichment analysis of DEGs in CTL vs. CTR and CSL vs. CSR. The top 20 significantly enriched pathways in each comparison are shown.

A total of 10,316 differentially expressed genes (DEGs) were identified across the four pairwise comparisons. Specifically, 7387 DEGs (3649 up-regulated and 3738 down-regulated) were detected in the CSL vs. CSR comparison, 7981 DEGs (4021 up-regulated and 3960 down-regulated) in CTL vs. CTR, 867 DEGs (308 up-regulated and 559 down-regulated) in CTL vs. CSL, and 1933 DEGs (777 up-regulated and 1156 down-regulated) in CTR vs. CSR (Figure 2a).

To identify genes responsive to freezing stress in walnuts, overlapping DEGs from the CSL vs. CSR and CTL vs. CTR comparisons were analyzed using a Venn diagram (Figure 2b). A total of 9611 freezing stress-responsive DEGs were obtained across the two varieties. Among these, 2853 up-regulated and 2880 down-regulated DEGs were commonly expressed in both varieties. Additionally, 24 DEGs exhibited opposite expression patterns between CS and CT under freezing stress.

Kyoto Encyclopedia of Genes and Genomes (KEGG) enrichment analysis was conducted to explore the key pathways involved in the freezing stress response in CS and CT. The top 20 significantly enriched pathways for each comparison are presented in Figure 2c. Notably, 15 pathways were commonly enriched in both varieties under freezing stress, including ‘ribosome’, ‘biosynthesis of amino acids’, ‘DNA replication’, ‘biosynthesis of secondary metabolites’, ‘mismatch repair’, ‘lysine biosynthesis’, ‘alanine, aspartate and glutamate metabolism’, ‘homologous recombination’, ‘circadian rhythm’, ‘glycolysis/gluconeogenesis’, ‘fructose and mannose metabolism’, ‘galactose metabolism’, ‘glycosphingolipid biosynthesis’, ‘α-linolenic acid metabolism’, and ‘flavonoid biosynthesis’.

### 2.3. Analysis of Genes Involved in Flavonoid Biosynthesis Under Freezing Stress

**Figure 3:**
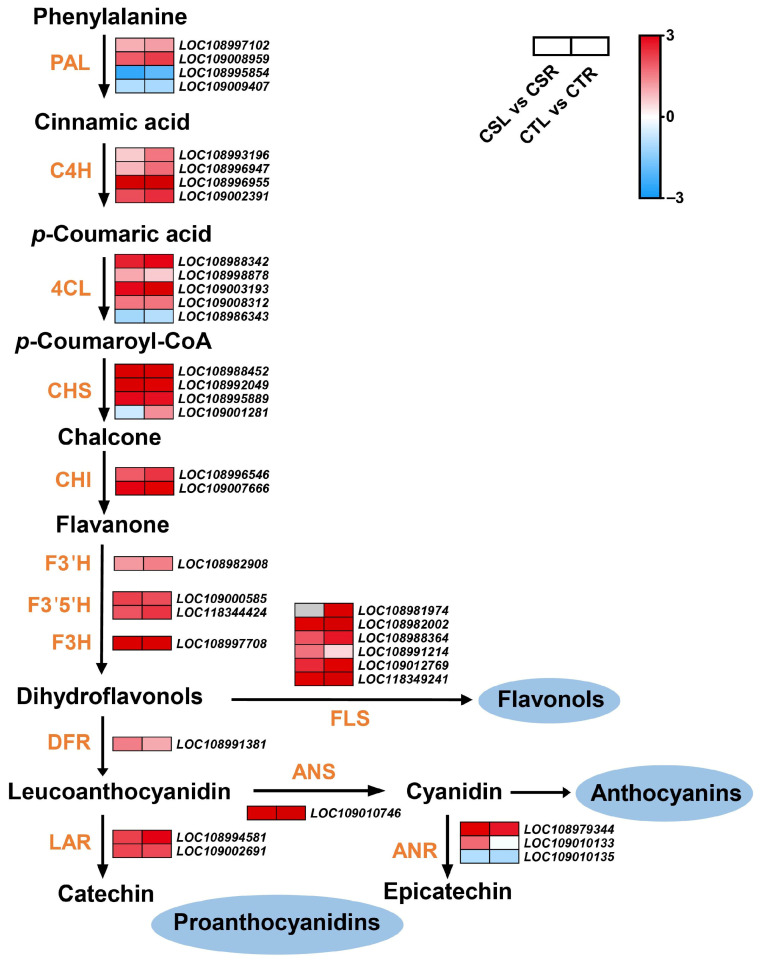
Expression profiles of differentially expressed genes (DEGs) involved in flavonoid biosynthesis under freezing stress. DEGs encoding key enzymes of the flavonoid biosynthesis pathway were mapped and analyzed in the cold-sensitive variety ‘Qingxiang’ (CS) and the cold-tolerant variety ‘Chandler’ (CT) under freezing treatment. Enzymes represented include: PAL, phenylalanine ammonia-lyase; C4H, trans-cinnamate 4-monooxygenase; 4CL, 4-coumarate-CoA ligase; CHS, chalcone synthase; CHI, chalcone isomerase; F3′H, flavonoid 3′-hydroxylase; F3′5′H, flavonoid 3′5′-hydroxylase; F3H, flavonoid 3-hydroxylase; FLS, flavonol synthase; DFR, dihydroflavonol 4-reductase; LAR, leucoanthocyanidin reductase; ANS, anthocyanidin synthase; ANR, anthocyanin reductase.

Flavonoids are well known to play critical roles in plant adaptation to low-temperature stress. Consistent with this, KEGG enrichment analysis showed that the flavonoid biosynthesis pathway was significantly enriched in both CS and CT under freezing stress. To further investigate the effects of freezing stress on flavonoid biosynthesis-related genes in walnuts, a simplified flavonoid biosynthesis pathway was constructed based on KEGG pathway analysis and gene function annotation (Figure 3). In total, 36 DEGs encoding 13 key enzymes were mapped into this pathway, including four genes encoding phenylalanine ammonia-lyase (PAL), four encoding trans-cinnamate 4-monooxygenase (C4H), five encoding 4-coumarate-CoA ligase (4CL), four encoding chalcone synthase (CHS), two encoding chalcone isomerase (CHI), one encoding flavonoid 3-hydroxylase (F3H), one encoding flavonoid 3′-monooxygenase (F3′H), two encoding flavonoid 3′5′-hydroxylase (F3′5′H), six encoding flavonol synthase (FLS), one encoding dihydroflavonol 4-reductase (DFR), two encoding leucoanthocyanidin reductase (LAR), one encoding anthocyanidin synthase (ANS), and three encoding anthocyanin reductase (ANR). Except for the down-regulation of two *PAL* genes (*LOC108995854* and *LOC109009407*), one *4CL* gene (*LOC108986343*), and one *ANR* gene (*LOC109010135*), all other DEGs involved in flavonoid biosynthesis were significantly up-regulated in at least one variety under freezing stress, indicating that enhanced flavonoid metabolism may contribute importantly to the freezing stress responses in walnuts.

### 2.4. Analysis of Transcription Factors Under Freezing Stress

**Figure 4:**
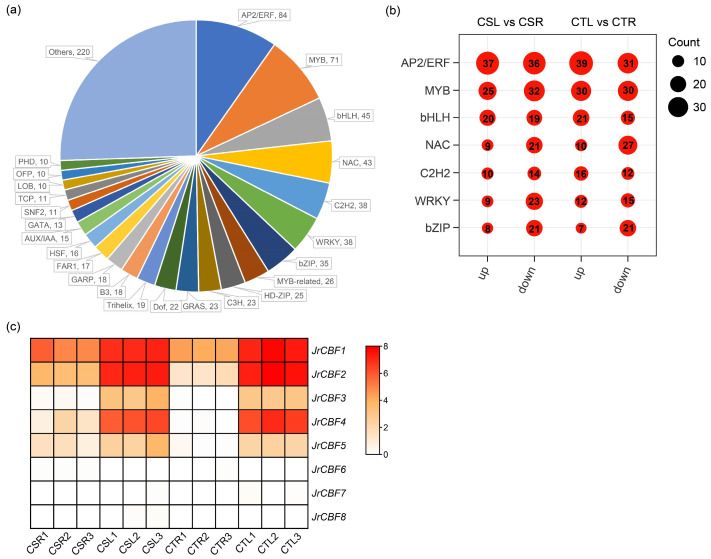
Identification and analysis of transcription factors responsive to freezing stress in walnuts. (**a**) Distribution of transcription factor (TF) families among the 861 freezing stress–responsive TFs identified. Only families containing more than 10 members are shown; (**b**) Numbers of up- and down-regulated genes within the seven largest TF families (AP2/ERF, MYB, bHLH, NAC, C2H2, WRKY, and bZIP) in the CSL vs. CSR and CTL vs. CTR comparisons; (**c**) Expression profiles of *JrCBF* genes under freezing stress. The heatmap is based on log-transformed FPKM values, with the color scale indicating relative expression levels.

Transcription factors (TFs) play pivotal roles in regulating plant responses to low-temperature stress. Among the 9611 freezing stress-responsive genes identified in this study, 861 were predicted to encode transcription factors using iTAK v1.7a software. These TFs were distributed across multiple families, with 24 families containing more than 10 members (Figure 4a). Notably, the AP2/ERF, MYB, bHLH, NAC, C2H2, WRKY, and bZIP families each comprised more than 30 members. The number of up-regulation and down-regulation genes within these seven TF families under freezing stress was further analyzed, suggesting that these families are involved in transcriptional regulation during freezing stress responses in both walnut varieties (Figure 4b). Among them, the AP2/ERF family contained the largest number of differentially expressed TFs. Importantly, the CBF subfamily, known as a central regulator of cold-responsive pathways, belongs to the AP2/ERF family. We therefore conducted a more detailed analysis of the walnut CBF gene family.

A total of eight *JrCBF* genes were identified in the walnut ‘Chandler’ genome and designated as *JrCBF1* to *JrCBF8* following a previous report (Table S2) [18]. The predicted proteins encoded by these genes range from 212 to 266 amino acids in length, with corresponding molecular weights ranging from 23.25 to 29.45 kDa. With the exception of JrCBF3, the isoelectric points (pI) of all other members are below 7, suggesting that the majority of these proteins are acidic. The instability index for all JrCBFs exceed 50, suggesting that they are unstable proteins. Moreover, all proteins exhibited negative grand average of hydropathicity (GRAVY) values, indicating hydrophilic properties.

The expression profiles of *JrCBFs* under freezing stress were further analyzed based on transcriptomic data (Figure 4c). *JrCBF1*, *JrCBF2*, *JrCBF3*, *JrCBF4*, and *JrCBF5* were significantly up-regulated under freezing stress in both walnut varieties, with *JrCBF1*, *JrCBF2*, and *JrCBF4* exhibiting the strongest induction. In contrast, *JrCBF6*, *JrCBF7*, and *JrCBF8* showed relatively low expression levels and no significant changes in response to freezing stress. These results indicate that *JrCBF1*, *JrCBF2*, and *JrCBF4* may act as key regulators of freezing tolerance in walnuts.

### 2.5. Weighted Gene Correlation Network Analysis of DEGs

**Figure 5:**
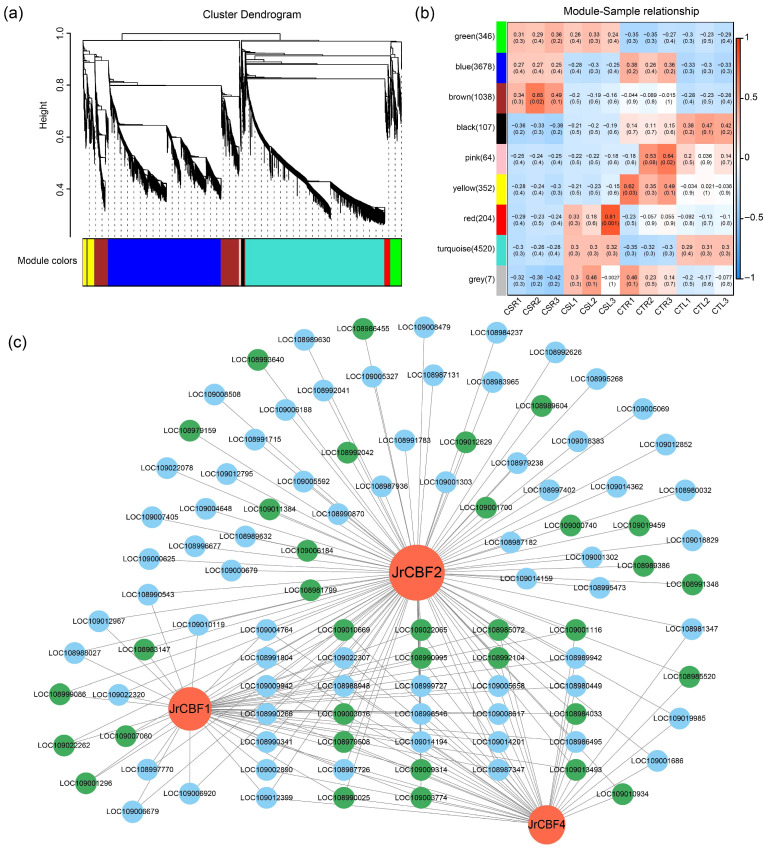
Weighted gene correlation network analysis (WGCNA) of differentially expressed genes. (**a**) Hierarchical clustering tree showing the co-expression modules identified by WGCNA; (**b**) Heatmap of correlations between co-expression modules and experimental samples. The left panel indicates the modules and the number of genes in each, while each column corresponds to a sample. Cells display the correlation coefficients between individual modules and samples; (**c**) Co-expression network of *JrCBF1*, *JrCBF2*, and *JrCBF4* within the turquoise module with edge weight greater than 0.65. Genes with CRT/DRE cis-elements in their promoter sequences are highlighted in green.

Weighted gene correlation network analysis (WGCNA) was employed to investigate the co-expression patterns of DEGs, and 10,316 DEGs were assigned to eight co-expression modules represented with different colors (Figure 5a). Each module comprises 107 to 4520 DEGs with genes displaying highly correlated expression profiles, while seven DEGs that did not show strong correlation with any module were grouped into the gray module (Figure 5b).

Among the TFs analyzed, *JrCBF1*, *JrCBF2*, and *JrCBF4* were strongly induced under freezing stress and may act as central regulatory factors in the walnut freezing stress response. Interestingly, all three *JrCBF* genes were assigned to the turquoise module. To further explore their regulatory network, a correlation network was constructed using an edge weight threshold of 0.65 (Figure 5c). A total of 102 DEGs showed a high correlation with these three *JrCBF* genes, among which 33 DEGs were commonly associated with all three *JrCBF* genes. CBF proteins are known to activate the expression of downstream genes by binding to the CRT/DRE (A/GCCGAC) cis-elements in promoter regions. Furthermore, we identified the promoter sequences of 102 co-expressed DEGs, among which 34 genes contained CRT/DRE motifs (labeled in green). Notably, these included two transcription factors, *JrGATA15* (*LOC108985072*) and *JrMYB308* (*LOC108990995*), which may serve as important secondary regulators in the CBF-mediated freezing response pathway.

### 2.6. RT-qPCR Validation

**Figure 6:**
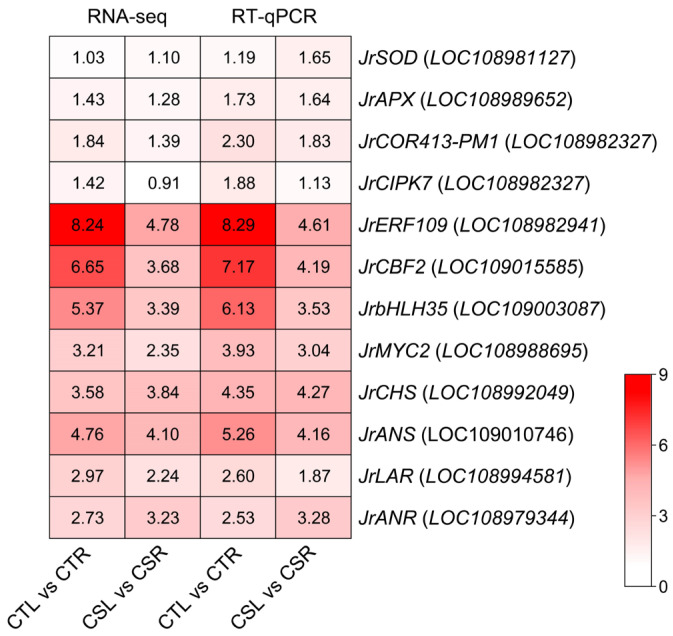
Validation of the RNA-seq data by real-time quantitative PCR (RT-qPCR) of twelve differentially expressed genes. The heatmap shows the log_2_ fold change values. For RT-qPCR analysis, each assay was performed with three biological replicates, and each biological replicate was analyzed with three technical replicates. *GAPDH* was used as the internal reference gene.

To verify the reliability of the RNA-seq data, 12 DEGs were randomly selected for real-time quantitative PCR (RT-qPCR) analysis, including genes encoding antioxidant enzymes (*JrSOD*, *JrAPX*), cold-responsive protein (*JrCOR413-PM1*), a protein kinase (*JrCIPK7*), transcription factors (*JrERF109*, *JrCBF2*, *JrbHLH35*, and *JrMYC2*), as well as four flavonoid biosynthesis genes (*JrCHS*, *JrANS*, *JrLAR*, *JrANR*). The expression patterns obtained from RT-qPCR were highly consistent with the RNA-seq results, thereby validating the accuracy and reliability of the transcriptomic dataset generated in this study (Figure 6).

## 3. Discussion

Spring frost is one of the most detrimental abiotic stresses that threatens walnut growth and productivity, resulting in substantial financial losses [24,27]. However, the molecular mechanisms governing walnut freezing tolerance have remained largely unexplored. In this study, comparative transcriptome analysis between cold-tolerant and cold-sensitive walnut varieties under freezing stress revealed extensive transcriptional reprogramming, with a total of 9611 DEGs identified, suggesting that walnuts respond to freezing stress through a complex molecular regulatory network. The large number of common DEGs, including 2853 upregulated and 2880 downregulated genes, highlights the presence of conserved molecular responses that are activated regardless of genetic background.

KEGG enrichment analysis demonstrated that multiple metabolic and signaling pathways are significantly involved in walnut responses to freezing stress, among which flavonoid biosynthesis emerged as a key pathway. Research has shown that the accumulation of secondary metabolites in plants plays a crucial role in the process of cold adaptation, particularly the activation of flavonoid metabolic pathways, which is of great significance in antioxidant defense [4,28]. Flavonoids can scavenge excess ROS by acting as antioxidants and alleviate oxidative stress damage to cells [28,29]. The construction of a simplified flavonoid biosynthesis pathway containing 36 DEGs encoding 13 key enzymes further supports the notion that flavonoid metabolism in walnuts is strongly activated under freezing conditions. This finding is consistent with a previous study on metabolomic responses of walnuts to chilling stress, where metabolites respond to cold stress mainly associated with phenylpropanoid biosynthesis, flavonoid biosynthesis, and phenylalanine metabolism [30]. Thus, the activation of flavonoid biosynthesis in walnut likely represents a crucial adaptive strategy to counteract freezing-induced oxidative damage.

CBF, also known as the dehydration-responsive element binding protein (DREB1), plays a critical role in the plant’s response to cold stress [31]. CBF transcription factors activate the expression of downstream *COR* genes by binding to CRT/DRE elements, enabling plants to adapt to cold stress. This mechanism has been well-characterized in species such as Arabidopsis [32], wheat [33], apples [34], and pomegranates [35]. Studies in walnuts have also contributed to our understanding of the CBF family. For instance, Han et al. identified five CBF family members in the ‘Chandler’ walnut genome and cloned the *JfDREB1A* from Xinjiang wild walnut, which was found to enhance cold tolerance of transgenic Arabidopsis [36]. Zhou et al. identified four *CBF* genes in the ‘Chandler’ genome and seven *CBF* genes in the ‘Zhongmucha-1’ walnut genome [37]. Additionally, Liu et al. identified eight *CBF* genes in the ‘Zhongmucha-1’ genome and found that *JrCBF1* and *JrCBF2* were significantly up-regulated under low-temperature stress [18]. In this study, we identified eight JrCBFs based on the new chromosome-scale assembly of the walnut reference genome (Chandler v2.0) [38], which is consistent with the findings of Liu et al. [18]. Further transcriptome analysis of two walnut varieties revealed that *JrCBF1*, *JrCBF2*, *JrCBF3*, *JrCBF4*, and *JrCBF5* were induced by freezing stress, with *JrCBF1*, *JrCBF2*, and *JrCBF4* showing particularly strong up-regulation. These findings suggest that these *JrCBFs* may serve as key regulatory factors in the walnut’s response to freezing stress.

To gain deeper insight into the regulatory network of these *JrCBFs*, we constructed a co-expression network using WGCNA, with *JrCBF1*, *JrCBF2*, and *JrCBF4* as core nodes. This network contained 102 highly correlated genes with *JrCBFs*, of which 34 genes contained CRT/DRE elements in their promoter regions. Notably, two transcription factors, *JrGATA15* and *JrMYB308*, were identified among these genes. Research has shown that the GATA transcription factor family plays crucial roles in plant growth, development, and response to abiotic stress. For example, *OsGATA16* has been shown to regulate cold tolerance in rice by inhibiting the expression of *OsWRKY45-1* [39]. In addition, the apple MYB domain 308-like gene *MdMYB308L* interacts with *MdbHLH33*, enhancing its binding to the promoters of *MdCBF2* and *MdDFR*, thereby positively regulating cold tolerance and anthocyanin accumulation in apples [40]. Given these findings, it is likely that *JrGATA15* and *JrMYB308* are involved in the walnut’s response to freezing stress. However, the specific roles of these transcription factors in walnut’s cold tolerance need further investigation.

**Figure 7:**
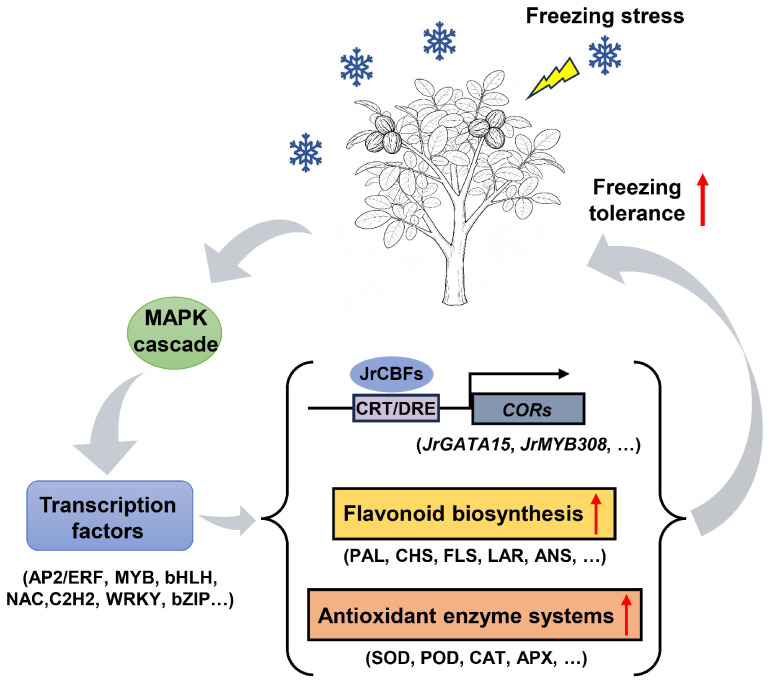
A simplified model of the walnut’s response to freezing stress.

Based on our findings, we propose a simplified model on how walnuts respond to freezing stress (Figure 7).

## 4. Materials and Methods

### 4.1. Plant Materials

Nine-year-old plants of the cold-sensitive variety ‘Qingxiang’ (CS) and the cold-tolerant variety ‘Chandler’ (CT) were used as experimental materials. All plans were grown in the walnut experimental field of the Industrial Crop Institute, Shanxi Agricultural University (37°14′ N, 111°47′ E), and were managed under standard cultivation practices, including routine irrigation, fertilization, and pruning as required.

### 4.2. Freezing Treatment and Sample Collection

New shoots of CS and CT with basically consistent growth were collected on 15 April 2024 and subjected to a freezing treatment (−2 °C, 40–50% relative humidity) for 12 h. Shoots maintained at room temperature (20 ± 1 °C) for 12 h served as controls. Three independent biological replicates were performed, with each replicate consisting of at least six individual shoots pooled together. All samples were immediately snap-frozen in liquid nitrogen and stored at −80 °C until RNA extraction.

### 4.3. Determination of Relative Electrical Conductivity

REC was measured using leaves from new shoots immediately after completion of the freezing or room temperature treatments. Leaves were rinsed with deionized water and cut into small segments (1–2 mm), and 0.1 g of tissue was placed in a centrifuge tube containing 10 mL of deionized water and soaked for 10 h. The initial conductivity (R1) was measured using a DDSJ-308A conductivity meter (INESA, Shanghai, China). The sample was then incubated in a boiling water bath for 30 min and cooled to room temperature, and the final conductivity (R2) was measured. The REC value was calculated as the ratio of R1 to R2, expressed as a percentage [41]. Three independent biological replicates were performed, and the significance of the difference among mean values was tested using Duncan’s multiple range test (*p* < 0.05). Data visualization was performed using Origin v8.5 software.

### 4.4. RNA Sequencing and Data Analysis

Transcriptome analyses were conducted by Wuhan Metware Biotechnology Co., Ltd. (Wuhan, China). The total RNA was extracted using the modified CTAB method, and a total of 12 RNA-seq libraries (three biological replicates per treatment) were constructed. After removing the adapter sequences and low-quality reads, clean reads were aligned to the walnut reference genome Chandler 2.0 by HISAT2 [38,42]. Gene expression levels were quantified as fragments per kilobase of transcript per million mapped reads (FPKM). Differential gene expression analysis was performed using the DESeq2 v1.22.1 R package [43]. The resulting *p*-values were adjusted according to the false discovery rate (FDR) method of Benjamini and Hochberg [44]. DEGs in pairwise comparisons were defined as those with |log2 fold change| ≥ 1 and FDR < 0.05.

Functional annotation of genes was performed using the KEGG, NCBI non-redundant protein sequences (NR), SWISS-PROT, and TrEMBL databases. Novel genes were predicted with StringTie [45]. TFs were predicted using the iTAK [46]. PCA, hierarchical clustering of samples, Venn diagram construction, KEGG enrichment analysis, and WGCNA were performed on the Metware Cloud platform (https://cloud.metware.cn, accessed on 13 February 2025). The gene co-expression network was visualized using Cytoscape v3.9.1 software. All heatmaps were generated using TBtools-II v2.34 software [47].

### 4.5. Genome-Wide Identification of CBF Genes in Walnut

Three Arabidopsis thaliana CBF protein sequences (AtCBF1, AtCBF2, AtCBF3) were used as queries to identify candidate CBF genes in the walnut reference genome Chandler 2.0 through BLASTP searches. Candidate protein sequences were further examined using the InterPro database (https://www.ebi.ac.uk/interpro, accessed 21 March 2025) to determine protein family classification. Proteins assigned to the dehydration-responsive element-binding protein 1A-I family (IPR045277) were considered members of the walnut CBF family. The physicochemical properties of the identified JrCBF proteins were analyzed using the Expasy-ProtParam tool (https://web.expasy.org/protparam, accessed on 21 March 2025).

### 4.6. Real-Time Quantitative PCR Analysis

Total RNA was extracted using the EasyPure^®^ Universal Plant Total RNA Kit (TransGen, Beijing, China). First-strand cDNA was synthesized from 1 μg of total RNA using HiScript II QRT SuperMix for qPCR (Vazyme, Nanjing, China). RT-qPCR assays were performed with ChamQ Blue Universal SYBR qPCR Master Mix (Vazyme, Nanjing, China) on an Applied Biosystems QuantStudio 1 Plus real-time PCR system (Thermo Fisher Scientific, Waltham, MA, USA). Relative gene expression levels were calculated using the 2^−ΔΔCT^ method [48]. Each assay included three biological replicates, and each biological replicate was analyzed with three technical replicates. *GAPDH* was used as the internal reference gene [49]. The primers for RT-qPCR are listed in Table S3.

## 5. Conclusions

This study provides a comprehensive analysis of the molecular mechanisms underlying walnut responses to freezing stress. By performing transcriptome analyses on cold-tolerant and cold-sensitive walnut varieties, a large number of common and unique DEGs were identified, underscoring the shared and divergent mechanisms underlying cold tolerance in walnuts. KEGG enrichment analysis revealed the flavonoid biosynthesis pathway being significantly activated, demonstrating the crucial role of flavonoids in walnut’s adaptation to freezing stress. In addition, WGCNA highlighted a co-expression network centered on the *JrCBF1*, *JrCBF2*, and *JrCBF4*, which are key regulators of freezing stress responses. This network also identified 34 genes that may interact with the *JrCBF* genes. Overall, this study provides valuable insights into the genetic and molecular responses of walnuts to freezing stress, particularly the activation of flavonoid biosynthesis and the role of *JrCBFs*. These findings lay a solid foundation for further research aimed at understanding and improving walnut cold tolerance.

## Data Availability

The raw sequence data reported in this paper have been deposited in the Genome Sequence Archive (GSA) in the National Genomics Data Center under accession number CRA029202 that are publicly accessible at https://ngdc.cncb.ac.cn/gsa (accessed on 27 August 2025).

## Supplementary Materials

The following supporting information can be downloaded at: https://www.mdpi.com/article/10.3390/plants14193089/s1, Table S1. Statistical analysis of RNA sequencing data; Table S2. Physicochemical properties of JrCBF proteins in walnut; Table S3. List of primer sequences for RT-qPCR in this study.
